# The mesolimbic reward pathway is necessary for disruptions in cocaine-seeking behavior following mediated devaluation

**DOI:** 10.1038/s41386-025-02119-x

**Published:** 2025-06-24

**Authors:** Bingxin Mo, Victoria K. Fex, Alice McQueney, Doris I. Olekanma, Christopher A. Reeves, Luciano S. Voutour, Sarah Simmons, A. J. Robison, Amy A. Arguello, Alexander W. Johnson

**Affiliations:** 1 Department of Psychology, Michigan State University, East Lansing, MI, USA; 2 Neuroscience Program, Michigan State University, East Lansing, MI, USA; 3 Department of Physiology, Michigan State University, East Lansing, MI, USA

**Keywords:** Learning and memory, Reward

## Abstract

We developed an approach to disrupt cocaine-seeking behaviors using mediated devaluation. Male rats underwent cocaine self-administration training in which active lever responses led to cocaine infusions and the presentation of a tone-light conditioned stimulus (CS). Subsequently, during mediated devaluation rats received non-contingent presentations of the cocaine-associated CS in a second distinct context, which led to the cue-evoked retrieval of associated memories. This was immediately followed by an intraperitoneal injection of lithium chloride (LiCl) and served to pair the memory of cocaine reward with gastric malaise. Consequently, this led to a substantial reduction in cocaine-seeking behavior during extinction training, relative to rats that received CS-saline or LiCl alone during mediated devaluation. Cue- and cocaine-evoked reinstatement testing indicated that the manipulations did not devalue the CS or the reinforcing properties of cocaine. A separate cohort of rats received a dual-viral chemogenetic strategy that permitted circuit-specific inactivation of midbrain ventral tegmental area (VTA) cells projecting to the nucleus accumbens (NAc). Inactivation of VTA→NAc circuitry during mediated devaluation prevented the subsequent reduction of cocaine-seeking behavior during extinction training. Overall, these findings suggest that intact mesolimbic signaling is required to enable disruptions in cocaine-seeking behavior following mediated devaluation.

## Introduction

Substance use disorders can be driven by the establishment of maladaptive learning and memory processes, endowing drug-related stimuli with the capacity to elicit substantial drug craving and relapse [1–3]. Furthermore, the transition from recreational to problematic substance use reflects a multifaceted learning process through which initial voluntary drug use spirals into compulsive drug seeking [4–6]. Thus, developing approaches that disrupt or devalue drug reward memories could be utilized for the treatment of substance use disorders.

In laboratory settings, rats trained to self-administer cocaine can develop rigid behaviors that are resistant to manipulations designed to attenuate cocaine-seeking. Earlier studies used outcome devaluation by pairing a cocaine-sucrose solution with the gastric malaise-evoking agent lithium chloride (LiCl) [7]. Although LiCl disrupted lever responding for a flavored sucrose solution, cocaine-sucrose responses were resistant to devaluation [7]. Alternatively, extinction has been used to devalue self-administration responding in rats using a seeking/taking chained schedule, in which extinction was carried out on the ‘taking’ lever [8]. This approach failed to disrupt cocaine-seeking in rats that received extensive self-administration training [8]. Similarly, an extensive history of cocaine-seeking leads rats to also develop compulsive drug-seeking, as evidenced by their continued responding for cocaine in the presence of an aversive conditioned stimulus (CS) that signaled foot shock [9].

The establishment of cocaine-seeking behaviors is thought to reflect a mesolimbic-dependent transition of control [5], whereby initial drug reinforcement is supported by increased dopamine expression originating from ventral tegmental area (VTA) cells that project to the nucleus accumbens (NAc). This reward pathway is particularly vulnerable to drug-induced molecular changes [10–12] and is thought to be critical in the development and strengthening of cocaine-associated memories [13–17]. Furthermore, the VTA→NAc pathway can promote the suppression of cocaine-seeking behavior [18, 19] in part through evoking aversive responses [20–22]. Accordingly, the mesolimbic circuit is critical for developing cocaine-associated memories and guiding drug-seeking behavior.

In the current study we examined whether mediated devaluation of cocaine could disrupt cocaine-seeking behavior—and, given its critical role in encoding the reinforcing properties of cocaine and establishing associated memories [2,11]—determined whether VTA→NAc circuitry was necessary for any attenuation in responding. Mediated devaluation was originally shown to successfully devalue food rewards and attenuate their intake [23, 24]. This phenomenon reflects the capacity for reward-paired CSs to activate features of the outcomes that they predict. When the retrieval of these associatively activated representations is immediately followed by LiCl, the outcome that was originally predicted by the CS is devalued [25–29]. We adapted this approach to examine whether mediated devaluation could disrupt cocaine-seeking behavior, perhaps by reshaping memories of cocaine reward [30]. Rats were trained to self-administer cocaine and during each infusion received the presentation of a tone-light CS. Subsequently, the CS was non-contingently presented in a second distinct context, followed by mediated devaluation via LiCl injections. We then examined the capacity for this devaluation approach to disrupt cocaine-seeking behavior during extinction training. This was followed-up with cue- and cocaine-induced reinstatement tests [31] that were designed to respectively determine whether mediated devaluation devalued the CS or the reinforcing properties of cocaine. Moreover, using a dual-viral intersectional strategy, we selectively inactivated VTA cells that project to the NAc during the CS-evoked retrieval phase and LiCl pairing, to determine the necessity of VTA→NAc circuitry for mediated devaluation. We expected mediated devaluation to disrupt future cocaine-seeking behavior in a manner that was dependent on mesolimbic circuitry.

## Materials and methods

### Animals

For electrophysiology studies, we used 4 male Sprague Dawley rats ~ 8 weeks of age, whereas for cocaine self-administration studies, 46 male 12-week-old Sprague Dawley rats were used. All rats were received from Envigo (Indianapolis, IN) approximately one week before beginning experiments and housed in the facility under a 12-hour reversed light cycle, with *ad libitum* food and water access. At 3 days post jugular catheterization, food pellets were restricted to 20 g per day to stabilize body weight. All procedures were approved by the Michigan State University Institutional Animal Care and Use Committee.

### Viral injection surgery

Rats were anesthetized with 4% isoflurane in oxygen and mounted into the stereotaxic apparatus. The subjects were bilaterally injected with a retrograde virus (0.25 µl per infusion) containing Cre-recombinase pENN.AAV.hSyn.HI.eGFP-Cre.WPRE.SV40 (Addgene, Watertown, MA; Plasmid #105540) in NAc and either Cre-dependent (Gi) DREADD (pAAV-hSyn-DIO-hM4D(Gi)-mCherry) (hM4Di) (Addgene; Plasmid #44362) or mCherry control (Addgene; Plasmid #114471) into the VTA. Viral constructs were injected via the following coordinates relative to Bregma: (i) NAc: AP 2.2 mm, ML ± 1.6 mm, DV −7 mm; AP 1.8 mm, ML ± 1.2 mm, DV −7.5 mm; and AP 1.8 mm, ML ± 0.75 mm, DV −7.5 mm. (ii) VTA: AP −5.4 mm, ML ± 0.7 mm, DV −7.5/−8.5 mm; and AP −6.2 mm, ML ± 0.7 mm, DV −7.5/−8.5 mm. Following stereotaxic surgery, all rats were single housed for the remainder of the study. To confirm viral targeting, rats were euthanized, and tissue stained using published methods [32]. Sections were stained for tyrosine hydroxylase (TH)-positive neurons in the VTA using AlexaFluor 488 (Thermo Fisher, Ann Arbor, MI) and colocalization with hM4Di mCherry-expressing cells was quantified. In the NAc, the density of EGFP-Cre was examined throughout core and shell subregions. We also examined the presence of hM4Di mCherry-positive fibers throughout the mesocorticolimbic circuit.

### Catheterization

Rats were anesthetized with a mixture of ketamine and xylazine cocktail (1 ml/kg), and a catheter was subcutaneously passed below the shoulder blade and inserted into the right jugular vein. Topical application of lidocaine and gentamycin were applied to suture areas, as previously described [33]. Following jugular catheterization, 10U heparin (0.1 ml) and the antibiotic cefazolin (0.1 ml) were used to flush catheters daily to maintain catheter patency. Intravenous (IV) infusion of propofol (0.1 ml) was administered in cases where catheter patency was uncertain.

### Drugs

Cocaine hydrocholoride was obtained from the NIDA Drug Supply System (Research Triangle Park, NC), dissolved in sterile saline. Active lever responses resulted in an IV infusion of 0.05 ml of cocaine (0.5 mg/kg per each infusion). For cocaine-primed cue-induced reinstatement, rats received a single intraperitoneal (IP) injection of cocaine at 5 mg/ml at a volume of 1 ml/kg. Clozapine-N-oxide (CNO) stock solution was made up by dissolving in 10% (2-Hydroxypropyl)-β-cyclodextrin and 0.2 M Phosphate-buffered saline (PBS). CNO working solution was prepared fresh daily and delivered by IP injection at a dose of 0.3 mg/kg. LiCl was dissolved into distilled water and provided at a volume of 5 ml/kg and concentration of 0.6 M.

### Electrophysiology

Allowing 4 weeks for steady viral expression, horizontal VTA-containing sections were prepared for ex vivo patch-clamp electrophysiology experiments. Recordings were performed on VTA slices expressing pAAV-hSyn-DIO-hM4D(Gi)-mCherry at baseline and following at least 15 min of 10 µM CNO application to confirm that CNO would disrupt neuronal excitation in VTA→NAc cells. Neuronal excitability in response to depolarization was assessed in whole-cell current-clamp mode on 4 neurons from 4 rats (1 neuron per rat). VTA neurons were given increasingly depolarizing current steps at 10 pA intervals ranging from +10 to +100 pA (5 s duration, with 20 s interstimulus intervals where cells were maintained at −70 mV between current depolarizing steps). Number of action potentials (APs) generated were recorded for each depolarizing step under baseline and CNO conditions.

### Self-administration training

All rats underwent self-administration (SA) training in operant chambers (Med Associates Inc., St. Albans, NY), equipped with two retractable levers and configured with diffuse contextual stimuli. The training context contained a continuous house light (0.4 fc brightness), pine-scented air freshener, and wire mesh flooring. Each session lasted 120 min (or a maximum of 70 infusions), during which time each active lever response led to the delivery of cocaine paired with the simultaneous presentation of a tone (80 dB, 1 kHz, 2 s on/off) and light (1.2 fc brightness, 2 s on/off) CS. Each infusion was followed by a 20 s timeout period, at which point lever responses resulted in no programmed consequences. Sessions continued until rat’s received > 10 infusions/daily within each 2-hour session for 10 days under a fixed-ratio 1 schedule.

### Mediated devaluation

Rats first received an IP injection of either CNO or vehicle 0.2 M PBS. Thirty minutes after this treatment, rats were placed into a second, alternate context: no houselight, slanted, black acrylic panel bisecting bar flooring, vanilla-scented air freshener. Rats were habituated to the new context for 6 min, and subsequently received ten presentations of the previously cocaine paired tone-light CS for 2 s, with each trial separated by a 30 s intertrial interval. Immediately afterwards, CNO-treated rats from each viral condition received an IP injection of either LiCl or 0.9% saline solution. This resulted in the following groups: mCherry-Saline (*n* = 9); mCherry-LiCl (*n* = 7); hM4Di-Saline (*n* = 7); hM4Di-LiCl (*n* = 9). To confirm that CNO itself did not impact mediated devaluation, an additional cohort of mCherry-LiCl (*n* = 4) rats treated with vehicle were included. In addition, a cohort of hM4Di-treated rats also received vehicle injections, a subset of which received mediated devaluation with LiCl as described above (paired group; *n* = 6), whereas the remainder received the same manipulations with the exception that no CS was presented (LiCl alone; *n* = 6). In this way, we could confirm whether pairing of CS-LiCl or LiCl alone influenced subsequent responding.

### Extinction

The data of critical interest for the study were collected 24 h after mediated devaluation. At this stage, rats received the first 2 h extinction training session in the previous cocaine-paired context, during which lever presses resulted in neither the infusion of cocaine nor presentation of the tone-light CS. These sessions were repeated each day until the subject performed fewer than 25 active lever presses for two consecutive sessions. The mean number of sessions to achieve this criterion were 7.06 ± 0.65 and did not differ across viral groups or treatment conditions.

### Reinstatement tests

Following extinction training, rats received a 2 h cue-induced reinstatement test, wherein each active lever response in the training context led to the delivery of the tone-light CS, followed by a 20 s timeout period. Subsequently, all rats underwent additional sessions of extinction, and after meeting criterion, received a cocaine-primed cue-induced induced reinstatement test. At this time, each subject received an IP injection of cocaine solution before placement into the chamber followed by an additional 2 h cue-induced reinstatement test.

### Data analysis

Electrophysiology results were examined using two-way current step (0–100 pA, at 10 pA intervals) X drug (baseline, CNO) repeated measures analysis of variance (ANOVA) to confirm CNO-evoked attenuation of APs in hM4Di expressing VTA→NAc cells. For immunofluorescent quantification we confirmed colocalization of TH+ and hM4Di mCherry expressing cells across the rostral-caudal axis of the VTA through a two-way condition (saline, LiCl) X depth (bregma −4.68 to −5.8, interval 0.2) ANOVA. For SA training, a three-way virus (mCherry, hM4Di) X condition (saline, LiCl) X session (1–10) ANOVA was conducted for active lever, inactive lever and infusions. The influence of mediated devaluation on active and inactive lever responding during the first extinction training session was examined by four-way virus X condition X time bins (1–6) X response (active, inactive) repeated measure ANOVA. The subsequent cue-induced reinstatement tests were determined by three-way virus X condition X time bins (1–6) repeated measure ANOVAs for each response. We confirmed reinstatement responding through phase (mean of previous two extinction sessions, reinstatement test) X condition X virus ANOVAs. The confirmation that CS-LiCl pairing were necessary for mediated devaluation was examined with a three-way condition (paired, LiCl alone) X time X response ANOVA. Significant interactions were followed up by ANOVAs and tests of simple main effects. Post-hoc comparisons were analyzed using Bonferroni tests.

## Results

### Inhibiting mesolimbic reward circuitry using dual-viral intersectional strategy

**Fig. 1:**
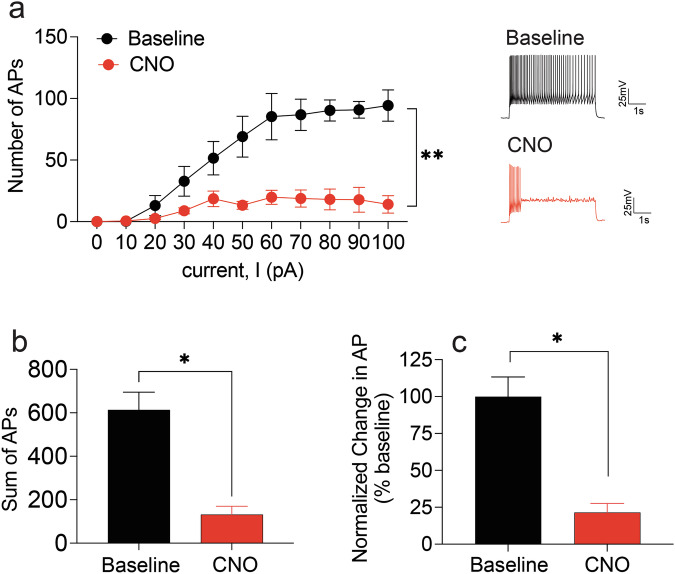
Whole cell patch clamp recordings in VTA→NAc hM4Di-expressing cells. **a** Bath application with CNO significantly attenuated the number of APs generated following step-wise increases in depolarization current (pA). **b**, **c** CNO treatment significantly reduced the (**b**) total number and (**c**) percentage increase in APs following the depolarization stimulus. ** significant current step X drug interaction, *p* < 0.01. *Overall reduction in APs, p’s<0.05.

In hM4Di VTA→NAc mCherry-expressing neurons, CNO treatment significantly reduced cell excitability (number of APs) across depolarizing current steps, with a main effect of CNO (F(1,33) = 106.6 *p* < 0.0001), current step (F(10,33) = 15.34 *p* < 0.0001) and significant interaction between the two variables (F(10,33) = 4.88, *p* < 0.01) (Fig. 1a). We also observed a significant reduction in the total number of action potentials following bath application with CNO (p’s<0.05) (Fig. 1b, c).

### Quantification of colocalized TH+ and hM4Di-mCherry expression

**Fig. 2:**
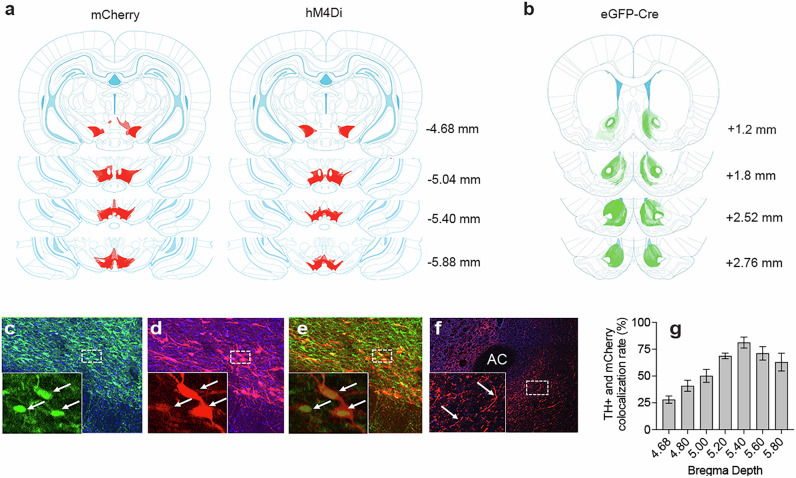
Immunohistochemical verification of Cre-dependent mCherry and hM4Di in VTA→NAc circuitry. **a** Heat maps revealing the extent of bilateral mCherry and hM4Di expression. **b** Heat maps revealing the extent of bilateral eGFP-Cre expression in NAc. Light shading reflects minimal spread, darker shading reflects maximal spread. **c**–**e** Colocalization between (**c**) TH+ neurons (green), (**d**) hM4Di (red) and (**e**) merged images. Arrows indicate somatic expression. **f** Representative micrographs of mCherry-hM4Di positive fibers in the NAc (arrows indicate visible fibers in NAc). **g** Quantification of colocalization between TH+ and hM4Di-expressing cells throughout the rostral-caudal axis of the VTA. Overall colocalization was ~ 64%. AC anterior commissure.

After excluding rats with no viral expression due to defective hM4Di virus (*n* = 10), we observed expression in the remaining rats throughout the rostral-caudal axis of the VTA (Fig. 2a). Quantification of the percentage of hM4Di-mCherry cells that were tyrosine hydroxylase positive (TH + ) revealed ~ 64% colocalization (Fig. 2c–e). This was similarly observed across mediated devaluation conditions (F < 1) and became more prominent as we extended caudally through the VTA (F (6,48) = 16.974; *p* < 0.0001) (Fig. 2g). We also examined the total number of TH+ cells that expressed hM4Di-mCherry, which revealed ~34% penetration of DREADDs in VTA TH+ cells. A brain wide analysis of hM4Di-mCherry fibers revealed the presence of immunoreactive fibers in the NAc (Fig. 2f), but not other VTA targets, including the amygdala, medial prefrontal cortex and hippocampus (Fig. S1). Finally, in the NAc, eGFP-Cre expression was broadly revealed throughout, with greatest density of expression noted in caudal NAc shell (Fig. 2b).

### Cocaine self-administration training

**Fig. 3:**
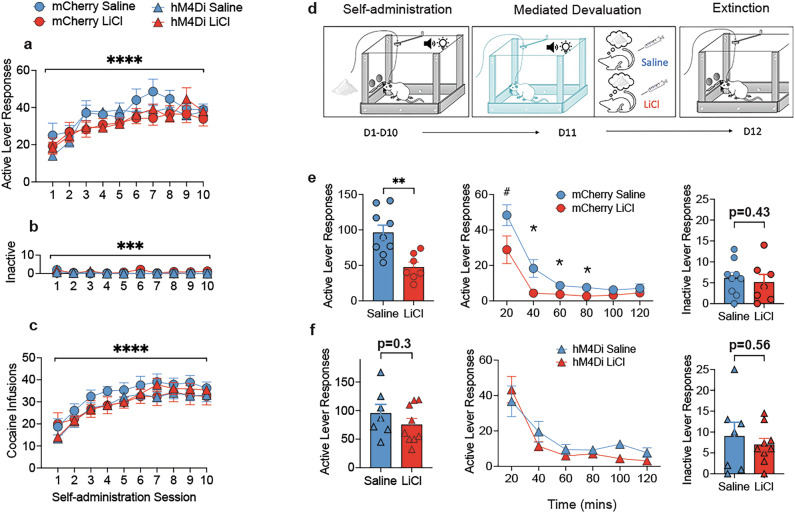
Cocaine self-administration training, mediated devaluation and extinction. **a** Active lever responses, **b** inactive lever responses, **c** number of infusions did not differ between viral or upcoming mediated devaluation conditions. Main effect of session, ****p < 0.0001, ***p < 0.001. **d** Simplified schematic for mediated devaluation testing adapted with permission [75]. **e** Active lever responses in the initial 2 h extinction training session in mCherry rats previously treated with either saline or LiCl during mediated devaluation. Significant reduction in cocaine-seeking behavior as a result of mediated devaluation, **p = 0.01, *p’s<0.05, #*p* = 0.06. Mediated devaluation did not impact inactive lever responses. **f** Inactivating VTA→NAc cells during mediated devaluation prevented the subsequent attenuation in cocaine-seeking behavior in hM4Di rats. Mediated devaluation did not impact inactive lever responses in hM4Di rats.

All rats similarly acquired self-administration, irrespective of viral condition (mCherry vs. hM4Di) or upcoming treatment during mediated devaluation (saline vs. LiCl) (Fig. 3a–c). Active lever responses gradually increased across self-administration sessions (F (9, 252)  =  23.95; *p* < 0.0001; Fig. 3a), which reflected a significant increase in responding from session 3 onwards relative to sessions 1 and 2 (p’s < 0.001). During the last 3 sessions of training, responses stabilized and did not differ significantly (p’s>0.13). In addition, inactive lever responses decreased similarly across conditions as training continued (F (9, 252) = 3.59, *p* < 0.001; Fig. 3b) and reflected higher inactive lever responding in sessions 1 and 2 relative to all other sessions (p’s <0.03). Finally, the number of cocaine infusions increased across sessions (F (9, 252)  =  47.773; *p* < 0.0001; Fig. 3c), remained stable by the last three sessions (p’s>0.27), and did not differ between viral or devaluation condition (p’s>0.57).

### Mediated devaluation disrupts cocaine-seeking behavior and requires VTA → NAc circuitry

Following cocaine self-administration training, mCherry and hM4Di treated rats received CNO prior to being placed into a distinct chamber where they received non-contingent presentations of the cocaine-associated CS. For approximately half the rats in each viral group, this was immediately followed by 0.6 M injection of LiCl, to elicit mediated devaluation, whereas the remaining rats received 0.9% saline injection (Fig. 3d). On the day following mediated devaluation to cocaine, rats were reintroduced to the previous cocaine-paired context. During the first extinction training session, the pattern of responding differed drastically as a function of viral group and mediated devaluation condition (Fig. 3e, f). ANOVA revealed a significant virus X condition X time interaction (F (5, 140) = 3.59, *p* = 0.004). In mCherry rats (Fig. 3e), for active lever responses, ANOVA revealed a main effect of condition (F (1,14) = 13.05, *p* < 0.01), time (F (5, 70) = 29.18, *p* < 0.001), and a tendency for an interaction between the two variables (F (5, 70) = 2.00, *p* = 0.08). These differences reflected a suppression of active lever responding in mCherry LiCl rats compared to mCherry saline at 40, 60 and 80 mins (F’s > 5.18; p’s <0.05; Fig. 3e) and a tendency for suppression during the first 20 mins (F (1,14) = 4.15; *p* = 0.06). Thus, mediated devaluation via LiCl led to substantial reductions in active lever responding in mCherry controls. By comparison, chemogenetic inactivation of VTA→NAc circuitry during mediated devaluation eliminated disruptions in active lever responding (Fig. 3f). In hM4Di rats, an analysis of responding during the first extinction training session revealed a main effect of time only (F(5,70) = 24.485; *p* < 0.001).

Additionally, when we examined responding between viral groups, for rats that received saline, ANOVA revealed a main effect of time only (F(5,70) = 25.833; *p* < 0.001), suggesting that silencing VTA→NAc circuitry alone did not disrupt active lever responding. However, for rats in the LiCl condition, we found an effect of time (F(5,70) = 28.126; *p* < 0.001) and a tendency for an effect of virus (F (1,14) = 3.922, *p* = 0.06). A comparison of responding between the two LiCl conditions (i.e., mCherry-LiCl, hM4Di-LiCl) revealed a main effect of viral group (F(1,5) = 5.46, *p* < 0.05), significant reductions in responding at 20–40 min in mCherry-LiCl compared to hM4Di-LiCl rats (F(1,14) = 6.50, *p* < 0.05), and a tendency for a reduction in active lever responses at 60–80 min (F(1,14) = 4.05, *p* = 0.06). Overall, these findings provide strong support that mediated devaluation can evoke substantial reductions in active lever responding, and intact mesolimbic reward signaling is necessary at the time of mediated devaluation to elicit reductions in responding. These latter findings did not reflect non-specific effects of CNO administration, as extinction test responding was similar in mCherry-LiCl rats treated with either vehicle or CNO prior to mediated devaluation (Figure S2; F < 1; *p* > 0.37). Finally, a comparison of inactive lever responses in mCherry and hM4Di rats revealed comparable responding across all time bins (F’s<3.9; p’s>0.07). (Fig. 3e, f; Figure S3).

### Mediated devaluation does not impact cue-induced or cocaine-primed reinstatement

**Fig. 4:**
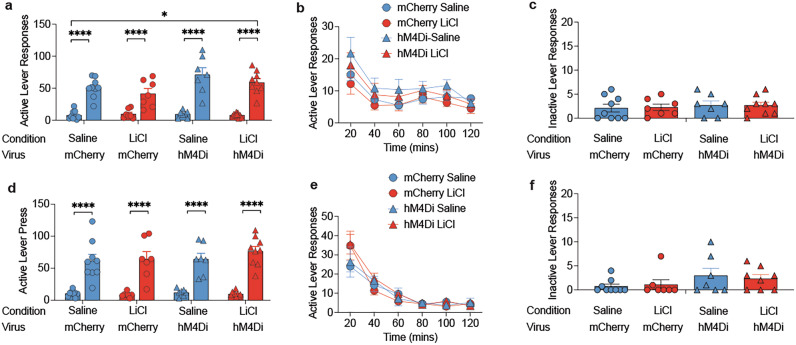
Cue and cocaine-primed reinstatement. **a** Cue-induced reinstatement led to substantial increases in active lever responses relative to previous extinction session. Overall hM4Di rats displayed greater reinstatement than mCherry group. **** main effect of testing phase, p’s<0.001. * main effect of virus, *p* < 0.05. **b** Time course of active lever responding during reinstatement test. **c** Overall inactive lever responses during reinstatement test. **d** Mediated devaluation did not impact cocaine-primed cue-induced reinstatement as similar levels of active lever responses were noted across saline and LiCl conditions. **** main effect of testing phase, p’s<0.001. **e** Time course of active lever responding during cocaine-primed reinstatement test. (**f**) Inactive lever responding during cocaine-primed reinstatement test.

We next examined whether directly pairing the CS with LiCl during mediated devaluation impacted cue-induced reinstatement behavior. Mediated devaluation did not influence the capacity of the CS to elicit active lever responses. Analysis revealed a main effect of testing phase (F (1, 28) = 155.56; *p* < 0.001), indicating that rats displayed CS-induced cocaine-seeking behavior relative to the two proceeding extinction sessions (Fig. 4a). However, mediated devaluation itself did not impact CS-induced responding (*p* = 0.1) (Fig. 4b, c). On the other hand, we observed a main effect of virus (F (1, 28) = 5.96; *p* < 0.05), indicating that prior inactivation of VTA→NAc circuitry may have protected against disruptions in encoding between the CS and cocaine that followed the cue exposure session during mediated devaluation. Mediated devaluation also appeared to have no effect on cocaine-primed reinstatement, ANOVA revealed a main effect of testing phase (F (1, 28) = 145.29; *p* < 0.001) (Fig. 4d–f) but no effect of virus or conditions (F’s <0.34; p’s>0.5).

### Mediated devaluation requires CS-LiCl pairings

**Fig. 5:**
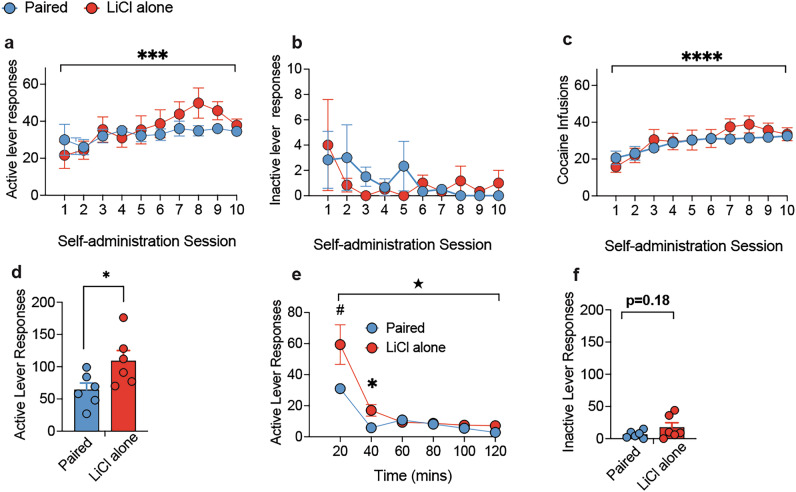
CS evoked retrieval of cocaine reward is necessary for mediated devaluation. **a** Active lever responses, **b** inactive lever responses, **c** number of infusions did not differ between upcoming mediated devaluation condition. Main effect of session, ***p = 0.001, ****p < 0.001. **d** Active lever responses in the 2 h extinction test in rats that received CS-LiCl pairing (paired) or LiCl alone (LiCl alone) during mediated devaluation. Significant reduction in cocaine-seeking behavior as a result of CS-LiCl pairing. Main effect of condition, *p < 0.05. **e** Time course of active lever responding during extinction test. ★Condition x time interaction (F(5,50) = 2.73, *p* < 0.05), reduction in cocaine-seeking as a result of mediated devaluation condition, #*p* = 0.05, *p < 0.05. **f** Inactive lever responses during extinction did not differ as a function of condition.

All rats similarly acquired self-administration, irrespective of upcoming mediated devaluation treatment (Fig. 5a–c). ANOVA revealed a main effect of session only for active lever responses (F(9,90) = 3.41, *p* = 0.001) and infusions (F(9,90) = 9.35, *p* < 0.0001). There were no significant effects or interaction for inactive lever responses (F’s<1.22; p’s>0.29). Following mediated devaluation, only rats that received CS-LiCl pairing displayed a reduction in active lever responding during the first extinction training session compared to rats that received LiCl alone (Fig. 5d–f). ANOVA revealed a main effect of condition (F(1,10) = 5.82, *p* < 0.05), time (F(5,50) = 31.3, *p* < 0.0001) and a significant condition X time interaction (F(5,50) = 2.73, *p* < 0.05). Analysis of responding within the extinction session revealed a tendency for reduced active lever responding at 20 mins (F(1,10) = 4.51, *p* = 0.05) and a significant reduction at 40 mins (F(1,10) = 7.45, *p* < 0.05). Inactive lever responses did not differ between the conditions (F(1,10) = 2.05, *p* = 0.18). These results suggest contingent pairing of the CS with LiCl (not LiCl alone) is necessary for mediated devaluation to disrupt active lever responses during initial extinction training.

## Discussion

Cocaine is highly addictive with significant abuse potential; however, despite extensive study there are no FDA approved treatments for cocaine use disorder (CUD). Accordingly, there is a significant need to develop approaches that can reduce cocaine use and relapse. By adapting a mediated devaluation approach, we were able to significantly disrupt cocaine-seeking behavior in rats with a history of cocaine self-administration. These findings are the first to demonstrate that mediated devaluation can attenuate instrumental reward-based performance and, moreover, identified that VTA→NAc cells were necessary at the time of mediated devaluation for this future disruption in cocaine-seeking behavior.

The VTA is composed predominantly of dopamine cells and consistent with known anatomical expression, around 60% of hM4Di-expressing cells that projected to the NAc, expressed dopamine [34]. The establishment of cocaine-seeking is supported by increasing dopamine release in the VTA→NAc pathway, which undergoes substantial molecular changes following cocaine exposure [10–12]. In addition to dopamine, VTA cells can also drive NAc activity through GABAergic and glutamatergic neurotransmission and have been shown to diminish drug-seeking behavior [18, 35]. The NAc also encodes and strengthens cocaine-associated memories [13–17]. Our results extend the role of the mesolimbic pathway in cocaine reward learning, and consistent with recent findings from our laboratory using food rewards [30], highlight the importance of this circuit for mediated devaluation.

Mediated devaluation was achieved through IP administration of LiCl, which is known to influence the actions of the mesolimbic reward system and can increase reward thresholds [36–40]. However, our findings cannot be attributed to the effects of LiCl alone on mesolimbic activity as administration of LiCl in the absence of CS pairings did not impact mediated devaluation. Alternatively, the actions of LiCl may reflect its capacity to influence memory consolidation processes. Substance use disorders can be viewed as maladaptive forms of learning and memory, whereby drug memories can invigorate the acquisition and relapse of drug-seeking behaviors [2, 3]. Indeed, many of the intracellular targets of LiCl are important for memory consolidation including, nitric oxide [41, 42], glycogen synthase kinase-3β [43, 44], and the extracellular signal-regulated kinase (ERK) [45]. Accordingly, it is possible that following CS-evoked retrieval, cocaine memories may have become destabilized by the subsequent administration of LiCl. However, LiCl-evoked disruptions in cue-associated cocaine memories are unlikely to account for the current results, given that unlike CS-evoked reconsolidation studies [46–48], mediated devaluation of cocaine left CS processing intact, at least as it relates to the capacity to reinstate cocaine-seeking behavior.

Our findings are perhaps best interpreted within an associative learning framework in which associatively activated event representations can substitute for the motivational events themselves, leading to the acquisition of new learning to previously encountered reward-related events [25, 49]. This has been demonstrated in mediated learning studies through which food aversions can be established when a CS-evoked representation of food is paired with LiCl-induced gastric illness [23, 50]. To account for these findings, it has been suggested that under certain circumstances, CSs can activate central nervous system processes that are typically evoked by food itself [51]. As such, when these representations are retrieved and followed by gastric malaise, a mediated devaluation to the food is established. Our findings suggest mediated devaluation can also occur to cocaine reward; however, the nature of this devaluation remains elusive. Under our testing conditions mediated devaluation did not impact the capacity of the CS to reinstate cocaine-seeking behavior, which consistent with food reward studies [23, 30, 50, 51] suggests processing of the CS itself was unaffected by LiCl pairings. Nevertheless, several differences in mediated devaluation of food and drug reward were revealed. One of the curious features of mediated devaluation with natural rewards is the transient nature of the phenomenon [25, 50]. That is, early in training CSs are posited to gain access to detailed reinforcement features that can substitute for the absent reward when LiCl is administered. However, this window of accessibility is thought to be limited, such that as training continues, the capacity for the CS to mediate a devaluation to the associated reward is lost [50, 51]. By contrast, the disruption in cocaine-seeking behavior that followed mediated devaluation occurred after extensive self-administration training, beyond that revealed in food reward studies. Moreover, when we compared cocaine-seeking behavior in rats that experienced either a high or low number of infusions (i.e., CS-cocaine pairings), surprisingly, rats that received more infusions displayed evidence of stronger mediated devaluation (Figure S4). Additionally, when a food associated CS is paired with LiCl, this can lead to the acquisition of a devaluation to the food reward. However, the results from the cocaine-primed cue-reinstatement test suggest mediated devaluation left intact the capacity for cocaine to reinstate drug-seeking behavior. Although it is possible that other approaches (e.g., reacquisition of self-administration responding) may have revealed evidence of cocaine reward devaluation, these findings suggest the reinforcing properties of cocaine were unaffected by mediated devaluation. Thus, the parameters underlying mediated devaluation to cocaine appear to differ from those controlling food reward.

It is possible that our approach resulted in mediated devaluation to the self-administration context, leading to an attenuation of active lever responding when rats were reintroduced to it. In this scenario, the CS evoked a representation of the self-administration context, which was subsequently associated with gastric malaise and correspondingly became devalued. Representations of the context are known to play a pivotal role in determining the nature of instrumental actions that guide performance [52–54]. Studies suggest that early on in training, actions (including drug-seeking behaviors) [4, 5, 55] can be guided by goal-directed response-outcome associations, such that responding is sensitive to the value of the acquired outcome [56–60]. With more extensive training, responding becomes habitual in nature and driven by stimulus-response actions that are insensitive to changes in outcome value [61, 62]. Interestingly, recent evidence points to the idea that performance can transition back from habitual to goal-directed control following manipulations to the context [52]. This raises the possibility that mediated devaluation to the context may elicit a switching of control from habit-based cocaine-seeking behavior [7] to a more goal-directed form of responding, which would be sensitive to reductions in outcome value following mediated devaluation. However, the merits of this account are currently difficult to reconcile with the lack of evidence of mediated devaluation to cocaine itself. Alternatively, some theorists have suggested that outcome representations can involve multiple parallel associations that independently encode sensory and motivational features of the outcome and evoke distinct consummatory and preparatory responses, respectively [63, 64]. Perhaps variations in the type of rewards used in mediated devaluation studies encourage differential encoding of these outcome properties. For instance, the use of food rewards might especially promote the formation of associations with detailed sensory features of reinforcers (e.g., sensory processing of taste), whereas drug rewards may drive encoding of more diffuse general motivational properties (e.g., prolonged psychoactive properties of cocaine) [63, 65]. As such, mediated devaluation in the current studies may have devalued these latter features of reinforcement, resulting in a reduction in cocaine-seeking behavior during initial extinction training.

Several limitations of the current study should be acknowledged. First, our chemogenetic approach targeted all ventral mesencephalic cell types projecting to the NAc. In addition to dopamine, the VTA contains both GABAergic and glutamatergic cells [66–68]. Thus, our approach had the potential to inhibit both inhibitory and excitatory signals into the NAc. Given the composition of the VTA and degree of hM4Di-TH colocalization, our findings likely reflect dopamine modulation. Nevertheless, the use of more targeted transgenic approaches (e.g., TH-Cre rat lines) [69] would provide additional clarity on the specific mesolimbic neurotransmitter systems influencing mediated devaluation of cocaine-seeking. Second, rats received CNO which can reverse metabolize to clozapine and disrupt signaling of the dopamine D2 receptor. However, it is unlikely that our studies reflect an impact of CNO per-se, as the concentration of this activator in the current study was lower than doses at which CNO is reverse-metabolized [70]. Moreover, mCherry rats that received vehicle or CNO displayed comparable mediated devaluation following CS-LiCl pairings. Third, it is also important to consider we adopted a hypertonic dose of LiCl that leaves open the possibility whether isotonic concentrations [71] might similarly reveal mediated devaluation. Forth, our study utilized male rats, however there are sex differences in the proclivity to self-administer cocaine [72], and therefore the extent to which mediated devaluation disrupts cocaine-seeking behavior in female rats should be explored.

Overall, our findings provide support for a novel approach to disrupt cocaine-seeking behavior using associative learning methodologies. These findings are consistent with the use of higher-order associative learning phenomenon as tools to disrupt drug-related learning [73, 74]. Moreover, they suggest that nestled within the mesolimbic circuitry, molecular mechanisms control the capacity to devalue the motivation to seek out cocaine following a history of self-administration, possibly by the encoding and reshaping of these memories from rewarding to aversive. As such, they offer great potential to develop future behavioral and pharmacological approaches to target CUD and other substance use disorders.

## Supplementary information

Supplemental Material

## Data Availability

The datasets generated during and/or analysed during the current study are available from the corresponding author on reasonable request.
