# Supplementary material for: The mesolimbic reward pathway is necessary for disruptions in cocaine-seeking behavior following mediated devaluation
Source: Neuropsychopharmacology. 2025 Jun 24;50(10):1515–23. doi: 10.1038/s41386-025-02119-x (PMC12339968; doi:10.1038/s41386-025-02119-x)
Supplement: Supplementary file 1 — Supplemental Material [file 41386_2025_2119_MOESM1_ESM.pdf]

Supplementary Materials and Methods

Figure S1

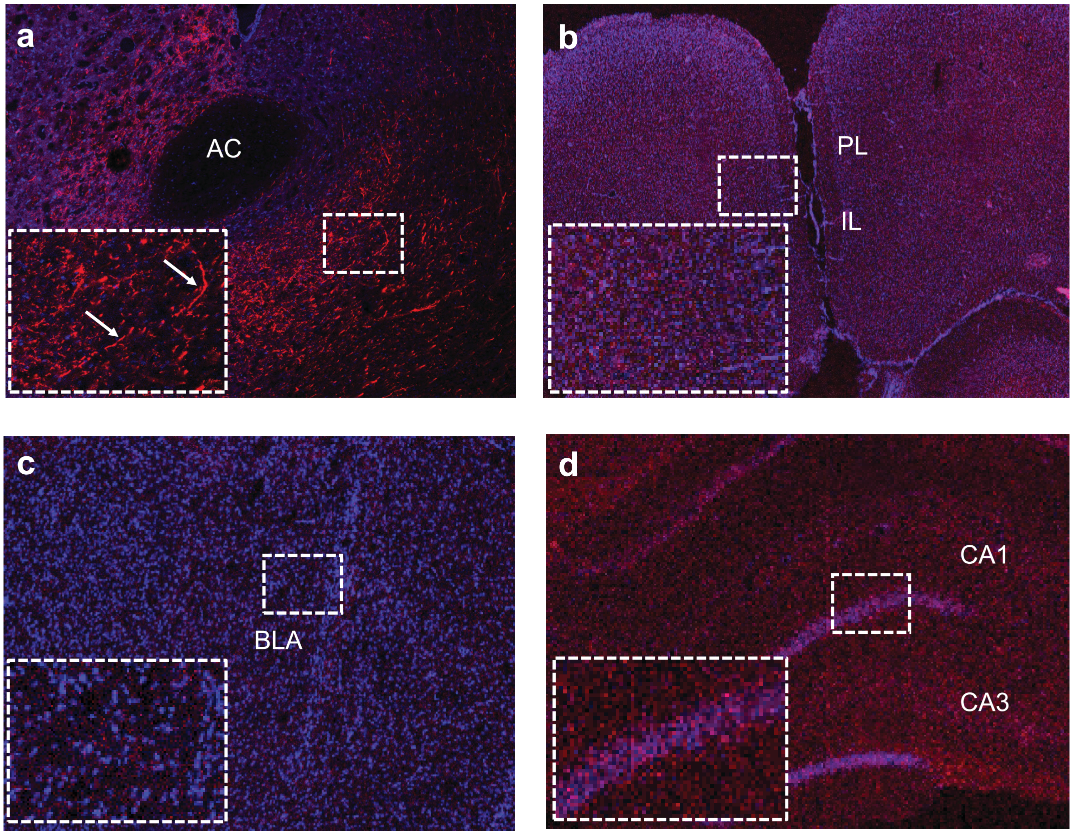

Figure S1. (a) Representative micrographs of mCherry-hM4Di positive fibers in the NAc (arrows indicate visible fibers in NAc). Other targets of the ventral mesencephalon did not display mCherry-hM4Di positive fibers, including **(b)** medial prefrontal cortex, **(c)** amygdala, and **(d)** dorsal hippocampus. Abbreviations: AC = anterior commissure, BLA = basolateral amygdala; CA1 = CA1 hippocampal field; CA3 = CA3 hippocampal field; IL = infralimbic cortex; PL = prelimbic cortex.

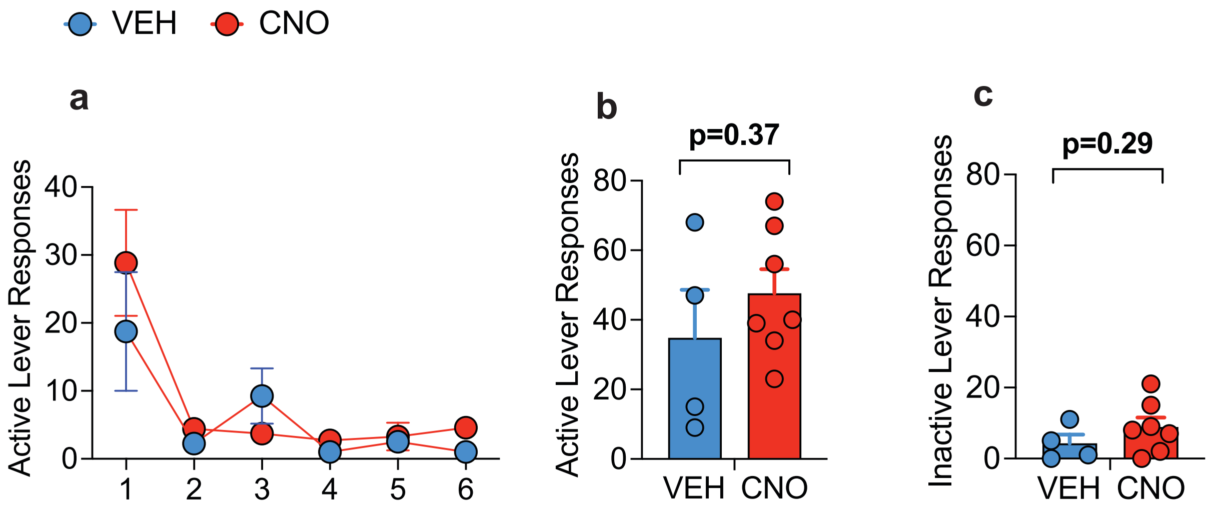

Figure S2. (a) Active lever responses in the 2 hr extinction test did not differ irrespective of whether mCherry rats received vehicle or CNO prior to CS-LiCl pairing. Two-way drug x time ANOVA revealed a main effect of time ($F(5,45) = 9.70$, $p<0.0001$), no effect of drug ($F<1$; $p>0.37$) or interaction between the variables ($F<1$; $p>0.51$). Overall **(b)** active and **(c)** inactive lever responses during the extinction test.

Figure S3

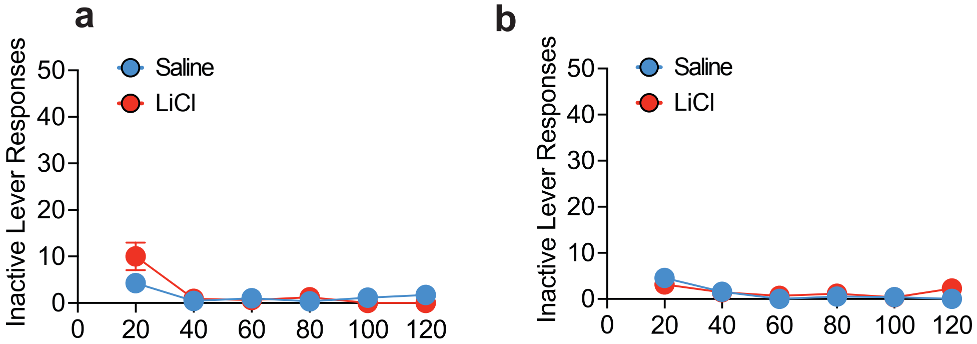

Figure S3. Inactive lever responses did not differ during the extinction test for **(a)** mCherry or **(b)** hM4Di rats irrespective of whether they were treated with LiCl. In mCherry rats, ANOVA revealed no differences in inactive lever responding between saline and LiCl groups at either time bin (Largest F-value; 120 min, $F(1,14) = 3.91$, $p=0.07$). Similarly, no differences in responding were seen in hM4Di-treated rats (Largest F-value; 120 min, $F(1,14).= 3.53$, $p=0.09$).

Figure S4

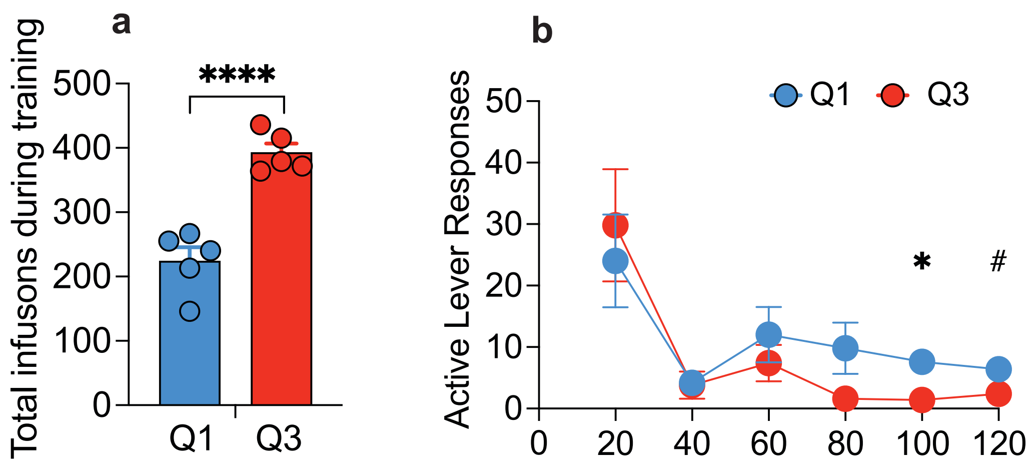

Figure S4. (a) Rats from groups mCherry veh-LiCl, hM4Di veh-LiCl, and mCherry CNO-LiCl were separated based on the total number of infusions attained during self-administration training. Rats that acquired the lowest number of cocaine infusions (Q1) were defined as those in the lower quartile of responders, whereas rats that highest number of cocaine infusions (Q3) were defined as those in the top 75th percentile of overall responders. ANOVA revealed a significant group difference in total infusions during training**** ($F(1,8) = 43.66$, $p=0.0001$). **(b)** Tendency for rats in Q3 to show stronger suppression of active-lever responding during the extinction test at 100 mins *($F(1,8) = 9.11$, $p=0.01$) and 120 mins #($F(1,8) = 4.19$, $p=0.07$.

Figure S5

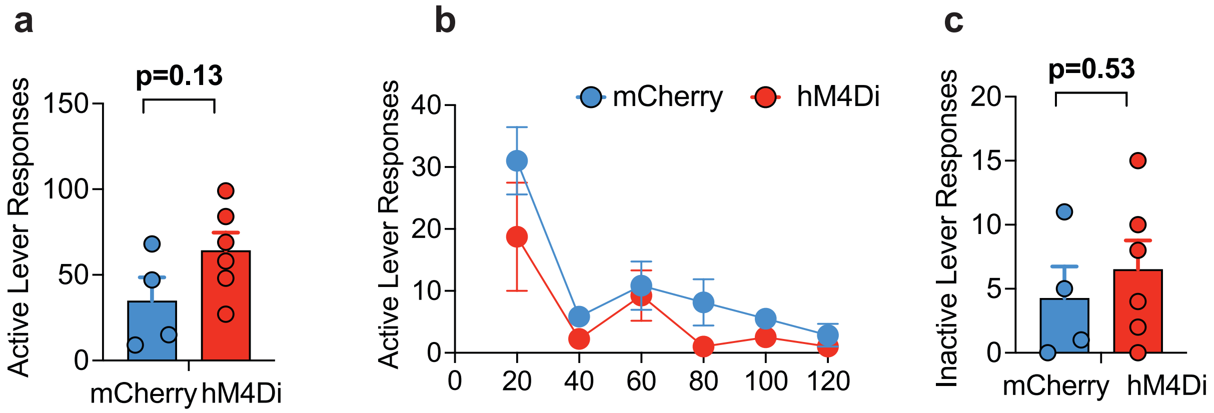

Figure S5. Expression of hM4Di alone did not impact extinction test responding following CS-LiCl mediated devaluation. mCherry-LiCl and hM4Di-LiCl rats treated with vehicle displayed comparable responding following mediated devaluation including **(a)** overall active lever responses, **(b)** active lever responses throughout the 2 hr extinction test, **(c)** and overall inactive lever responses. Analyses revealed no main effect of virus virus ($F(1,8) = 2.95$, $p=0.13$) nor virus x time bin interaction ($F<1$; $p>0.6$).
